# Supplementary material for: Notch Signalling Plays a Role in Patterning the Ventral Mesoderm During Early Embryogenesis in Drosophila melanogaster
Source: Int J Mol Sci. 2026 Jan 27;27(3):1284. doi: 10.3390/ijms27031284 (PMC12898112; doi:10.3390/ijms27031284)
Supplement: Supplementary file 1 [file ijms-27-01284-s001.zip › ijms-3922274-supplementary.pdf]

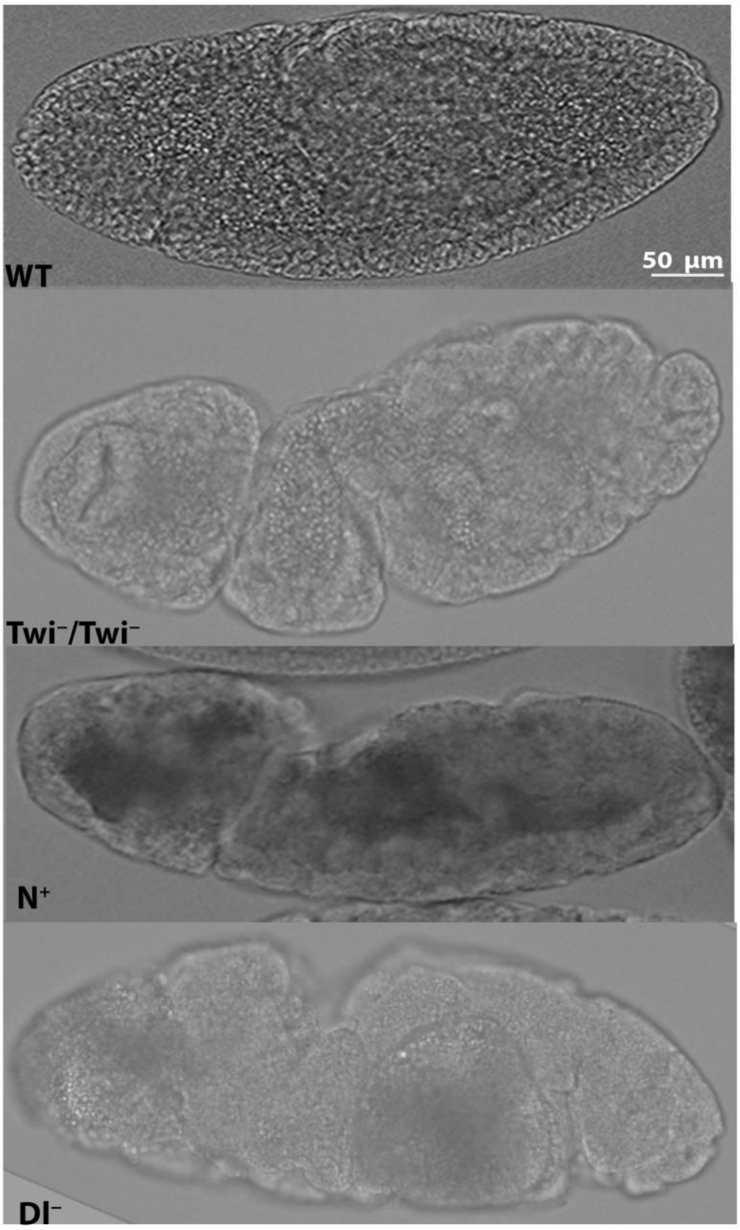

Supplementary figure S1. Morphological defects observed in Delta^{-} and N^{+} mutants are similar to those of Twist^{-} mutants. Bright field images of stage 9-11 wild type embryo (top), loss-of-function Twist mutant (2nd image), OptoNotch mutant (3rd image) and Delta^{-}mutant (4th image). Loss-of-function Twist mutants (Twi^{-}/Twi^{-}) have similar morphological defects as gain-of-function and loss-of-function mutants in the Notch signalling pathway such as aberrant invaginations along the AP axis and the overall twisted body phenotype. N=10 embryos/ genotype

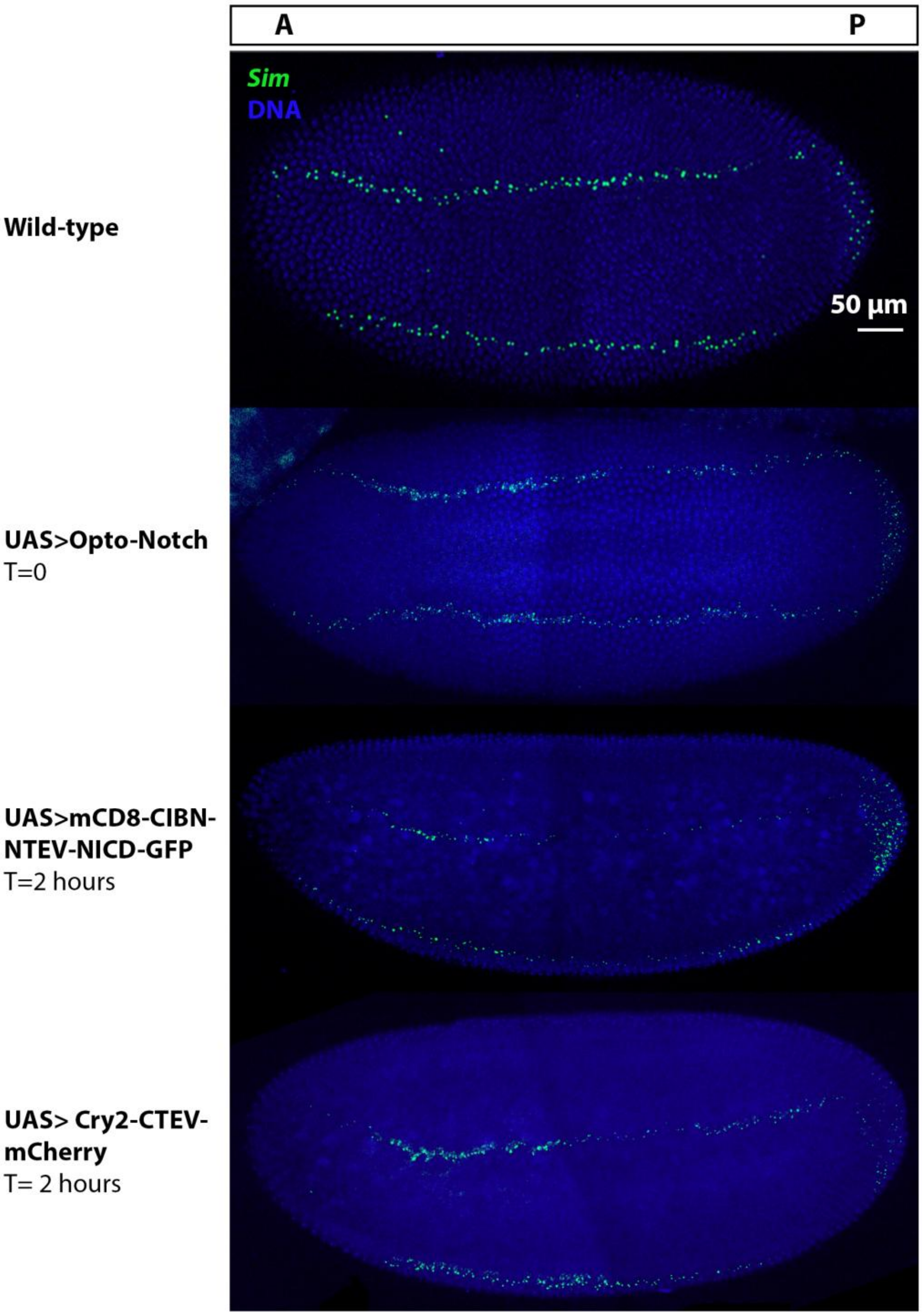

Supplementary figure S2. *Sim* mRNA expression does not vary between wild-type, non-photoactivated OptoNotch and control OptoNotch embryos. Fluorescent *in situ* hybridizations showing the expression of *sim* (green) in stage 5-6 embryos that; 1) do not express any transgenes (wild-type), 2) express OptoNotch but not photoactivated, 3) express only mCD8-CIBN-NTEV-NICD-GFP and photoactivated for 2 hours continuously, and 4) express only Cry2-CTEV-mCherry and photoactivated continuously for 2 hours. In embryos of all genotypes, *Sim* is expressed in the two rows of mesectoderm cells flanking the mesoderm and cells in the posterior end as in wildtype embryos. N=10 embryos/ genotype

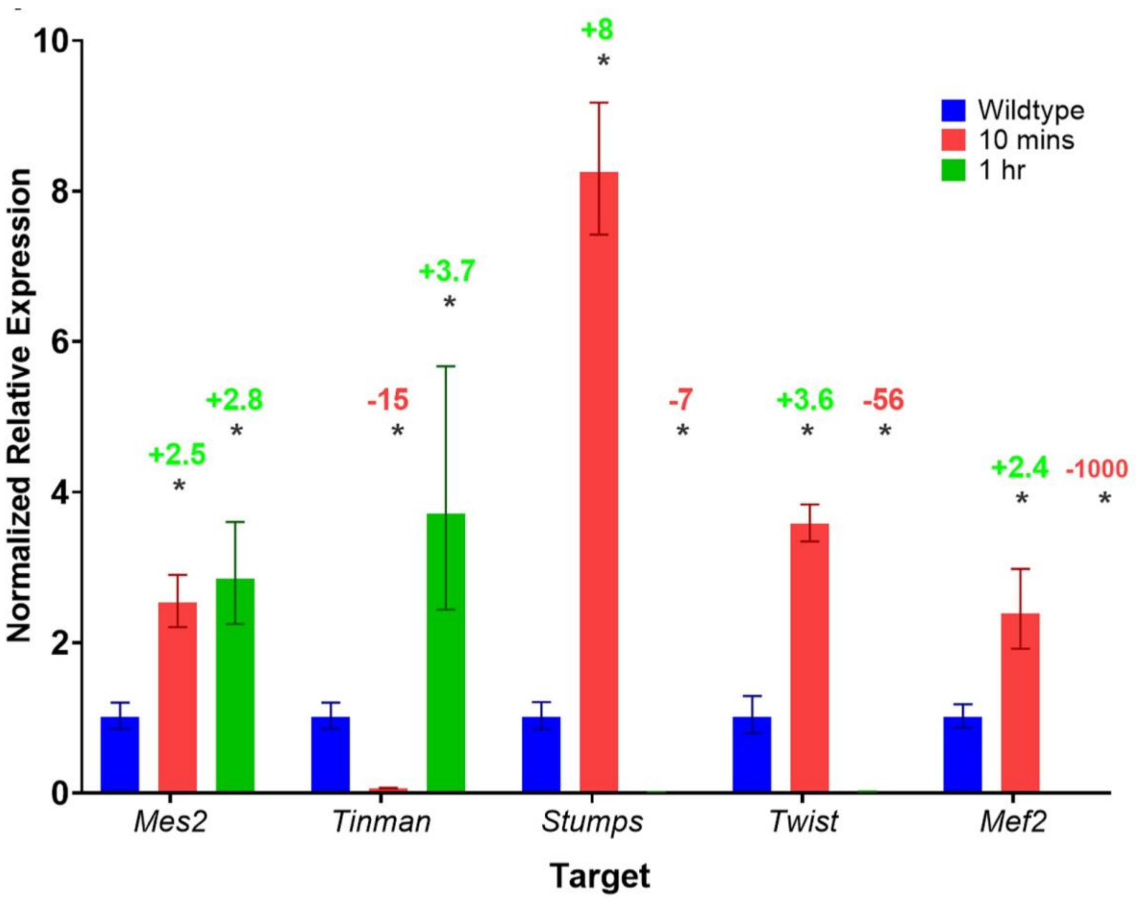

Supplementary figure S3. Expression of mesodermal genes in wild-type and OptoNotch embryos following photoactivation for short durations. qRT-PCR generated Normalized relative mRNA expression of mesodermal genes, n= 30 embryos at cephalic furrow formation in each genotype. Error bars represent SEM. Gene expression in wild-type (blue bars), OptoNotch embryos photoactivated for 10 minutes (red bars) and 1 hour (green bars) are shown. Short-term activation (10 minutes of photoactivation) of Notch signalling results in upregulation of *Mes2*, *Stumps*, *Twist*, and *Mef2*. Long-term (1 hour of photoactivation) activation of Notch signalling results in downregulation of *Stumps*, *Twist*, and *Mef2* but upregulation of *Mes2*. Upregulation of *Tinman* expression is only observed after long-term activation of Notch signalling (after 1 hour of photoactivation). A One-way ANOVA and a Tukey HSD test were performed to determine statistical significance. The statistical results are listed here. *Mes2*: ANOVA $F_{(3,8)}=69.7468$: $p=1.90x10^{-08}$, *Tinman*: $F_{(3,8)}=69.5154$: $p=1.94x10^{-08}$, *Stumps:* $F_{(3,8)}=207.0422$: $p=3.27x10^{-11}$, *Twist:* $F_{(3,8)}=388.3925$: $p=7.80x10^{-13}$, *Mef2*: $F_{(3,8)}=870.837$: $p=6.33x10^{-15}$. Statistical significance of the difference between the experimental group and the control group (WT) is denoted by '*', indicating Tukey HSD p-value <0.05 for that pairwise comparison. Only significant differences between experimental groups and WT are shown for simplicity.

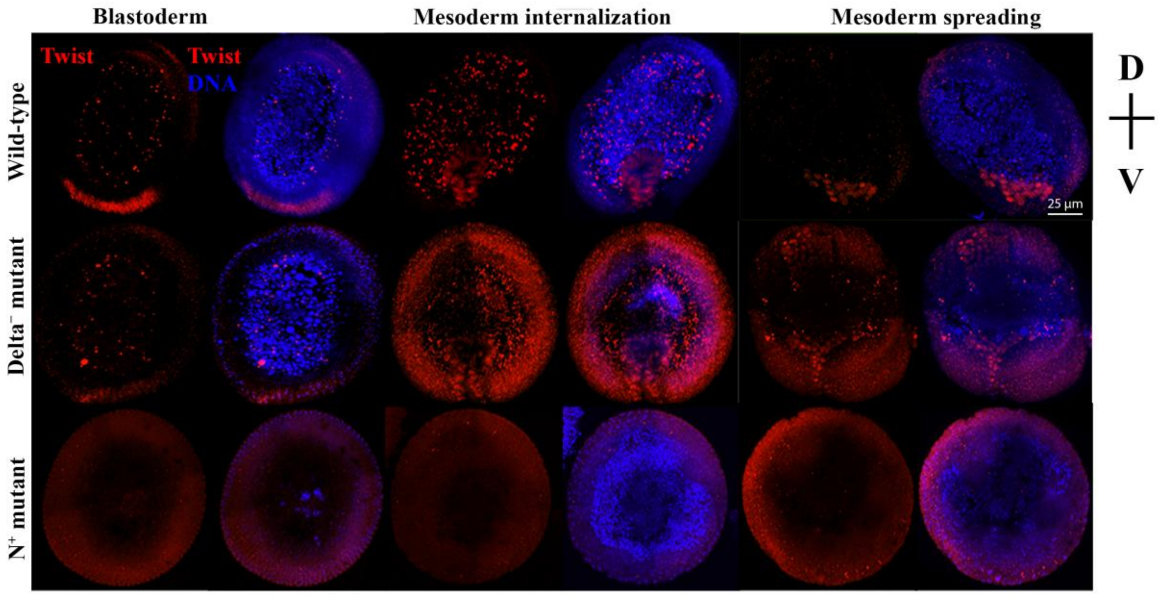

Supplementary figure S4. Cross-sections of embryos at different stages of gastrulation showing the decrease in Twist expression in Delta⁻ and OptoNotch (N⁺) mutants compared to wild-type embryos. Twist expression is decreased in loss-of-function and gain-of-function mutants compared to wild-type embryos during all the stages of gastrulation examined; cellularization, mesoderm internalization, and mesoderm spreading. The ventral side of embryos are at the bottom of each image. Although Twist expression is decreased, internalization and spreading of the mesoderm does not appear to be significantly impacted in mutant embryos except that in N⁺ embryos, there are additional groups of cells on the dorsal side of the embryo that are internalized. N=10 embryos/ genotype

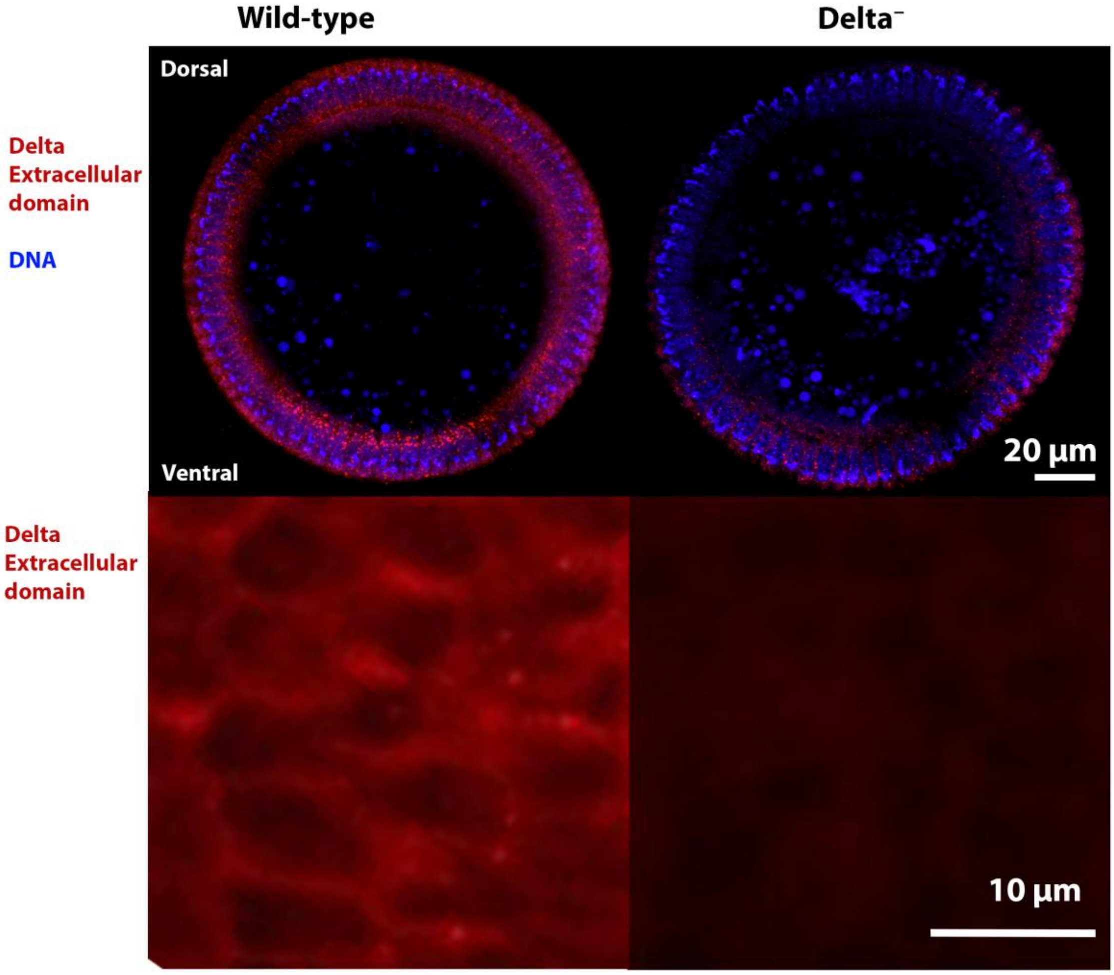

Supplementary figure S5. Heat-sensitive Delta mutation inhibits endocytosis of Delta when heat shocked at 32 °C, making it inaccessible for Notch extracellular domain and thereby inhibiting Notch signalling activation. A) Cross-sections of blastoderm wild-type and Delta⁻ embryos showing the decrease of Delta extracellular domain on cell membranes and intracellularly in Delta⁻ mutants compared wild-type embryos. B) Delta extracellular domain (red) expression in the ventral side of stage 6 wild-type and heat-shocked Delta⁻ mutant embryos. Delta extracellular domain is localized to cell membranes and inside the cells in wild-type embryos but is absent from most membranes of mutant embryos and no intracellular Delta is detected.

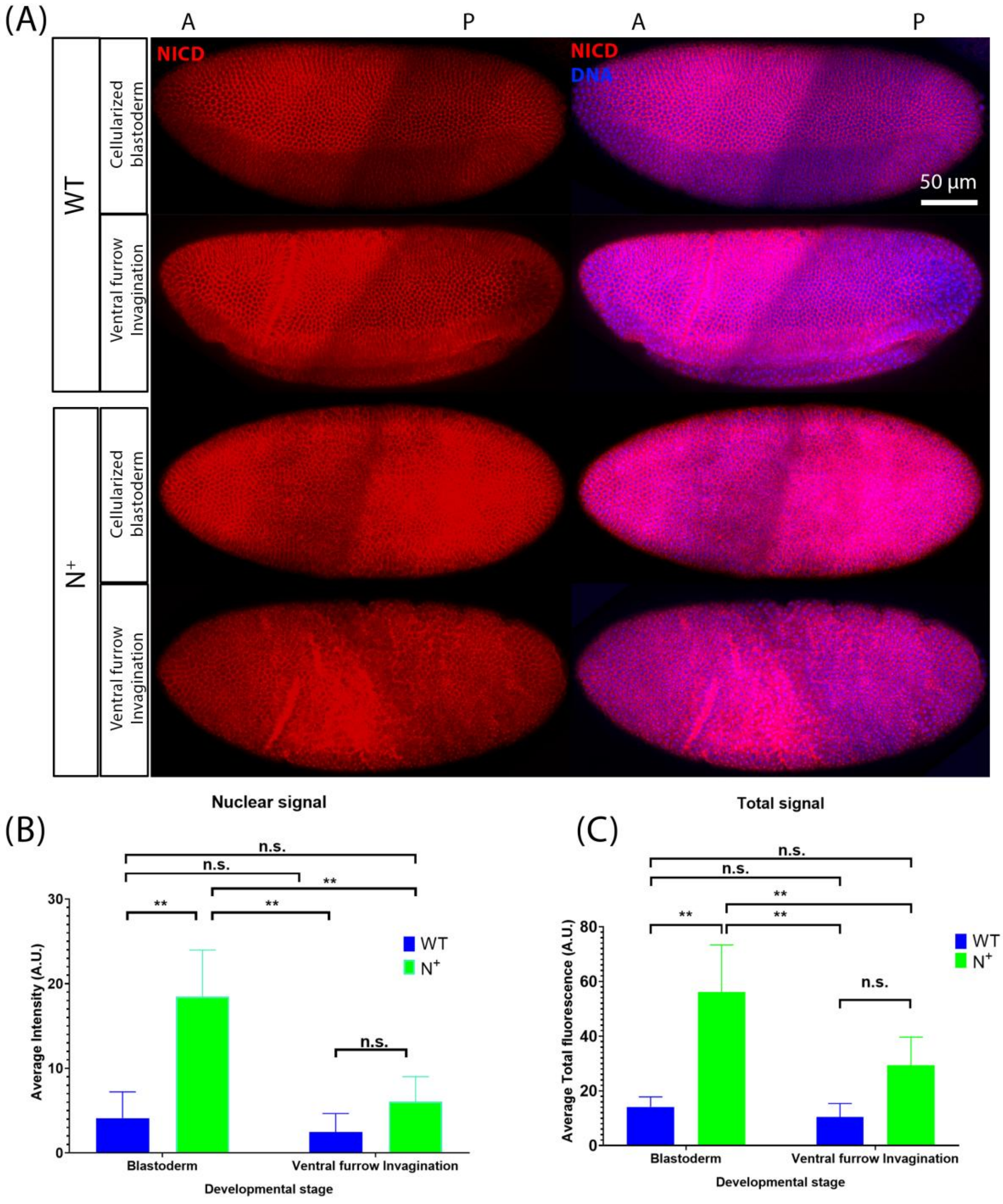

Supplementary figure S6. Expression of the intracellular domain of Notch (NICD) in wild-type (WT) and OptoNotch (N⁺) embryos photoactivated for 120 minutes. A) The expression of NICD is shown in red and nuclei are shown in blue in wild-type and OptoNotch embryos at different stages of embryogenesis. In wild-type cellularized blastoderms, NICD expression was detected at high levels on the plasma membrane of ectodermal cells and at lower levels in mesodermal cell membranes. NICD distribution is similar in wild-type embryos undergoing mesoderm internalization (ventral furrow formation). OptoNotch embryos showed high levels of NICD (red) across the entire embryo. B) N⁺ cellularized blastoderm embryos (N= 5) showed higher levels of nuclear NICD and total NICD levels (C) compared to their wild-type counterparts (N= 6). The expression of NICD in N⁺ embryos undergoing ventral furrow invagination (N= 4) was not statistically significantly different from their wild-type counterparts (N=4). A One-way ANOVA and a Tukey HSD test were performed to determine

statistical significance. Statistical significance is denoted by '**', indicating Tukey HSD p-value <0.01 for that pairwise comparison and 'n.s.' denotes a statistically non-significant difference, p>0.05. ANOVA for nuclear signal: $F_{(3,15)}$= 18.4278; p=2.72x10^{-5}. Tukey HSD: blastoderm WT vs N$^+$ p=0.0010053, blastoderm WT vs ventral furrow invagination WT p=0.8999947, ventral furrow invagination WT vs N$^+$ p=0.539361, blastoderm N$^+$ vs ventral furrow invagination N$^+$ p=0.0010053, blastoderm N$^+$ vs. ventral furrow invagination WT p=0.0010053, blastoderm WT vs ventral furrow invagination N$^+$ p=0.8397514. ANOVA for total signal: $F_{(3,15)}$=19.2096; p=2.14x10^{-5}. Tukey HSD: blastoderm WT vs N$^+$ p=0.0010053, blastoderm WT vs ventral furrow invagination WT p=0.8999947, ventral furrow invagination WT vs N$^+$ p=0.0920234, blastoderm N$^+$ vs ventral furrow invagination N$^+$ p=0.0083692, blastoderm N$^+$ vs. ventral furrow invagination WT p=0.0010053, blastoderm WT vs ventral furrow invagination N$^+$ p=0.1565156.

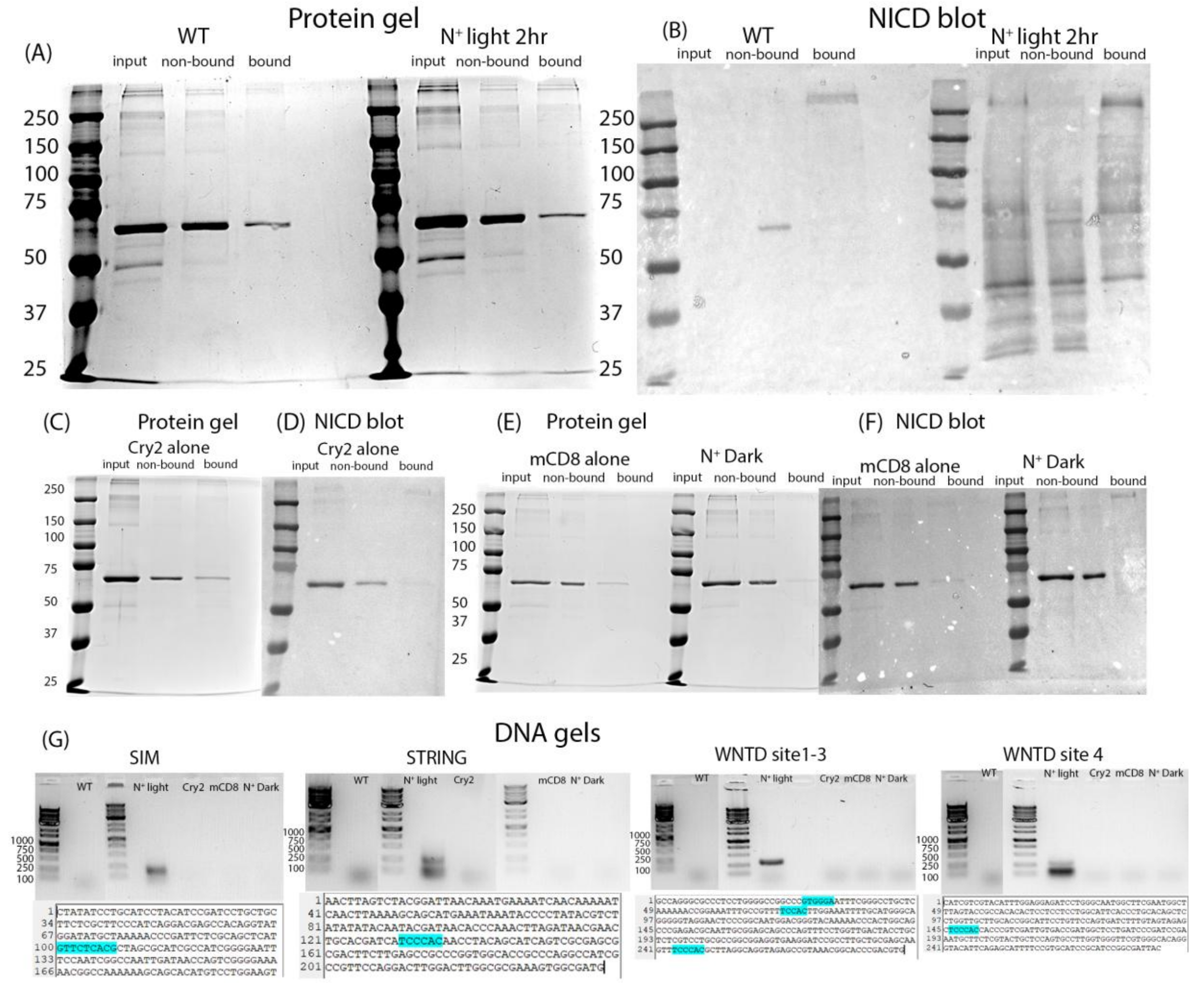

Supplementary Figure S7. ChIP-PCR analysis showing the detection of STRING and WNTD gene regions in OptoNotch-associated DNA. A) Coomassie-stained SDS-PAGE gel showing the input, non-bound, and bound fractions immunoprecipitated from stage 5-6 wild-type (WT) embryos and OptoNotch embryos photoactivated for 2 hours (N$^+$ light) using GFP-trap beads. B) Western blot against Notch intracellular domain (NICD) showing the detection of the full-length Notch protein at 300 kDa in the wild-type bound fraction and all fractions of N$^+$ light embryos. All fractions isolated from N$^+$ light embryos show additional bands from ~40-150kDa, which correspond to the size of NICD-GFP and subsequent proteolytic products. C) Coomassie-stained SDS-PAGE gel showing the input, non-bound, and bound fractions immunoprecipitated from stage 5-6 embryos expressing Cry2-CTEV-mCherry and photoactivated for 2 hours (Cry2 alone) using GFP-trap beads. D) Western blot against Notch intracellular domain (NICD) showing the detection of the full-length Notch protein at 300 kDa in the bound fraction of embryos expressing Cry2 alone. No bands were detected at 150 kDa in the bound fraction. E) Coomassie-stained SDS-PAGE gel showing the input, non-bound, and bound fractions immunoprecipitated from stage 5-6 embryos expressing mCD8-CIBN-NTEV-NICD-GFP (mCD8 alone) and OptoNotch embryos that were not photoactivated (N$^+$ Dark). F) Western blot against Notch intracellular domain (NICD) showing the detection of the full-length Notch protein at 300 kDa in the bound fraction of embryos expressing mCD8 alone and N$^+$ Dark embryos. No bands were detected at 150 kDa in the bound fraction of either group. G) DNA gels showing PCR results for regions of SIM, STRING, and WNTD containing Su(H) binding sites. DNA isolated from the GFP-trap beads bound fraction from each group of embryos was used as the template DNA. Bands at the expected sizes of ~200 bp were detected only in the photoactivated N$^+$ embryos, and no bands were detected

in bound DNA isolated from wild-type, Cry2 alone, mCD8 alone, or N$^+$ Dark embryos. Sequencing results corresponding to the detected amplicons are shown below each gel, where Su(H) binding sites are highlighted in blue.

Supplementary Table S1. Flylines used and generated in this paper.

Name in this paper	Source	Genotype
Wild-type (W1118)	Bloomington Stock Center (#3605)	w[1118]
Mat- alpha- tublin> Gal4	Bloomington Stock Center (#80361)	y[1] w[*]; P{matalpha4-GAL-VP16}67; P{matalpha4-GAL-VP16}15
UASp> Cry2-CTEV-mCherry	This paper	Chr3: UAS>Cry2-CTEV-mCherry / TM3
UASp>mCD8-CIBN-NTEV-NICD-GFP	This paper	Chr2: UAS> mCD8-CIBN-NTEV-NICD-GFP/ Cyo
UASp>OptoNICD	This paper	X: UAS> mCD8-CIBN-NTEV-NICD/ FM7i
Delta-TS	Kind gift by Eric Wieschaus	Chr3: DlRF/Dl6B37
Twi-	Bloomington Stock Center (#2381)	cn[1] twi[1] bw[1] speck[1]/CyO
OptoNICD	This paper	y[1] w[*]: UAS> mCD8-CIBN-NTEV-NICD-GFP; UAS>Cry2-CTEV-mCherry

Supplementary Table S2. Primer sequences used for cloning.

**Primer name**	**Sequence (5′-3′)**
Cry2 forward	CACCATGAAGATGGACAAAAAGACT
mCherry reverse	GCATGGACGAGCTGTACAAG
CTEV forward	ATCTGGGTAAGATCTGGTATGTCTAG
CTEV reverse	CTTGTACAGCTCGTCCATGC
Cry2 forward with pUASP homology	GATCAGATCCGCGGCCGCCACCATGAAGATGGACAAAAAGACT
mCherry reverse with homology to CTEV	ATCTTACCCAGATCTTGTACAGCTCGTCCATGC
CTEV forward with homology to mCherry	CGAGCTGTACAAGATCTGGGTAAGATCTGGTATGTCTAG
CTEV reverse with pUASP homology	TTAACGTTAACGTTCGAGGTCGACTCTAGACTAATTCATGAGTTGAGTCGCTTCC

Supplementary Table S3. Primer sequences for qRT-PCR for mesodermal target genes.

**Target gene**	**5′-3′ sequence**	**Source of the sequence**	**Annealing Temperature according to temperature gradient (°C)**
Sim Forward	CAGACGCTGGACGGATTCATC	https://www.flyrnai.org/flyprimerbank	59.0
Sim Reverse	AGCGACAAAATGGCATTCATCT		
Asph Forward	GGCGTTCACATTACAAAGATGGG	https://www.flyrnai.org/flyprimerbank	59.0
Asph Reverse	CCGACAGAGGAGTGTCCAGAT		

Tinman Forward	AATCCCCACCAGTACCTCAAC	https://www.flyrnai.org/flyprimerbank	**61.4**
Tinman Reverse	GGCAAGAGTGGCGATAGGG		
Traf4 Forward	CTCAACGCCTGTAAGCACGAT	This paper	57.1
Traf4 Reverse	GTGCAGGTGTACTGGAGATGA		
Twist Forward	CCTCAACGACGCCTTCAAG	https://www.flyrnai.org/flyprimerbank	**57.0**
Twist Reverse	AAGTCAATGTATCTTGTGGCCAG		
Mes2 Forward	CGCCCACGTCAAGCTAATG	https://www.flyrnai.org/flyprimerbank	**55.7**
Mes2 Reverse	GCCCTTTCGTTCTCTGATGACA		
Mef2 Forward	ATATCACGCATCACCGATGAAC	https://www.flyrnai.org/flyprimerbank	**61.4**
Mef2 Reverse	GGCGTACTGGTACAGCTTGT		
Neurotactin Forward	AAATCTCGCCCAGGACTG	https://www.flyrnai.org/flyprimerbank	**57.1**
Neurotactin Reverse	GCTGTGGCTTCAGACTTG		
NetrinA Forward	TGGGGACAACGTAACCTTGAC	https://www.flyrnai.org/flyprimerbank	**65.0**
NetrinA Reverse	GTAGATCACCATCGAATCGGGA		
Heartless Forward	TGGGAGCCAGGAGTCAAAAC	https://www.flyrnai.org/flyprimerbank	61.4
Heartless Reverse	GAACTGACATTCACCGGCAA		
WntD Forward	AGACAACATGATTTTTGCCATC	This paper	65.0
WntD Reverse	GGGTGTACTGGTAGTAGCTCA		
String Forward	GAAAACAACTGCAGCATGGAT	https://www.flyrnai.org/flyprimerbank	**64.5**
String Reverse	CGACAGCTCCTCCTGGTC		
Stumps Forward	ACTTTCCTGAAGAACCACTTCG	https://www.flyrnai.org/flyprimerbank	**64.5**
Stumps Reverse	AAGGGACCATCGTCAATTTTGAT		
Snail Forward	AGCCGCAGGATCTATCCCT	https://www.flyrnai.org/flyprimerbank	60.6
Snail Reverse	GTGTTTTTGAAAGGTTCAGCACA		

Supplementary Table S4. cDNA clones and primers used to amplify DNA templates to synthesize DIG and Biotin labeled antisense RNA probes for *in situ hybridizations*

Gene name	CG#	Clone#	Plasmid and antibiotic resistance	Primer sequences (5′-3′)	DGRC RRID and stock number
sim	CG7771	RE54280	pFlc-1	Forward: AATCATGTACATCTCGG<br>Reverse:<br>AACTGCTCGAGCAGCTATTCG	RRID:DGRC_93<br>45<br>Stock#: 9345
WntD (Wnt8)	CG8458	RE57705	PFLC-1 (ampR)	Forward:<br>CATGATTTTTGCCATCACATTC<br>Reverse:<br>GCATTCCTGTTCCAGTGATCTT	RRID:DGRC_97<br>48<br>Stock#: 9748
string	CG1395	LD47579	pOT2 (chlorR)	Forward:<br>TCAACAAAATGAGTGGATCTCG<br>Reverse:<br>GATCAGCGAGTTTAGGCCACT	RRID:DGRC_26<br>23<br>Stock #: 2623
netrinA	CG18657	RE11206	PFLC-1 (ampR)	Forward:<br>CCAAAGTAATTGGTCGCGATAC<br>Reverse:<br>GCAGCGGCACTCCAGGT	RRID:DGRC_26<br>23<br>Stock#: 2623
mes2	CG1110	SD09884	pOT2 (chlorR)	Forward:<br>CATACTCATGCCAACGTCTCAT<br>Reverse:<br>ACTCAATATCCACCGAAAAACG	RRID:DGRC_34<br>03<br>Stock #: 3403
Neurotactin	CG9704	LD22004	pOT2 (chlorR)	Forward:<br>CCGACTTCGTTTCGTGTTTAGT<br>Reverse:<br>AGTCCTTCGATCTCAGACTTGC	RRID:DGRC_57<br>36<br>Stock#: 5736
mef2	CG1429	LD08608	pBS SK - (ampR)	Forward:<br>CAGCATCAACGGCAGCAACA<br>Reverse:<br>ACTATTACCGTAGGTCTG	RRID:DGRC_44<br>09<br>Stock #: 4409
heartless (htl)	CG7223	LD32130	pOT2 (chlorR)	Forward:<br>AGCACGGATTACTATCGGAAGA<br>Reverse:<br>TACACCACTTCTGCAGGTTGTC	RRID:DGRC_52<br>17<br>Stock#: 5217
Stumps	CG31317	RE42507	pFlc-1 (ampR)	Forward:<br>CATCCCAACGACACATATTGTAATC<br>Reverse: TTCATGCTTAGCGGCATGC	RRID:DGRC_90<br>29<br>Stock #: 9029

Supplementary Table S5. Primer sequences used for ChIP-PCR analysis.

Gene name	Number of sites within amplicon	Sequence 5′-3′
**SIM**	**1**	**Forward:** CACTTCCAGGACATGTGC **Reverse:** CAGCGCCTATACCCTATATCC
**STRING** (region #1)	**1**	**Forward:** TACAACGAGGCCTATCGC **Reverse:** TCGTGTGCGAGAAACTTAGTC
**WNTD** (region #1)	**3**	**Forward:** TCATTCATGAGCACGTCGG **Reverse:** TGCAAATCCCAAGCCAGG
**WNTD** (region #2)	**1**	**Forward:** TGTGCCAATGGAGTAATCG **Reverse:** TAGTTGGATGGCATCGTC
